# Deep Learning Algorithms for Human Activity Recognition in Manual Material Handling Tasks

**DOI:** 10.3390/s25216705

**Published:** 2025-11-02

**Authors:** Giulia Bassani, Carlo Alberto Avizzano, Alessandro Filippeschi

**Affiliations:** 1 Institute of Mechanical Intelligence, Scuola Superiore Sant’Anna, 56124 Pisa, Italyalessandro.filippeschi@santannapisa.it (A.F.); 2 Department of Excellence in Robotics and AI, Scuola Superiore Sant’Anna, 56124 Pisa, Italy

**Keywords:** autoencoder, convolutional neural network (CNN), human activity recognition (HAR), manual material handling (MMH), recurrent neural network (RNN), wearable sensor network (WSN)

## Abstract

Human Activity Recognition (HAR) is widely used for healthcare, but few works focus on Manual Material Handling (MMH) activities, despite their diffusion and impact on the workers’ health. We propose four Deep Learning algorithms for HAR in MMH: Bidirectional Long Short-Term Memory (BiLSTM), Sparse Denoising Autoencoder (Sp-DAE), Recurrent Sp-DAE, and Recurrent Convolutional Neural Network (RCNN). We explored different hyperparameter combinations to maximize the classification performance (F1-score,) using wearable sensors’ data gathered from 14 subjects. We investigated the best three-parameter combinations for each network using the full dataset to select the two best-performing networks, which were then compared using 14 datasets with increasing subject numerosity, 70–30% split, and Leave-One-Subject-Out (LOSO) validation, to evaluate whether they may perform better with a larger dataset. The benchmarking network DeepConvLSTM was tested on the full dataset. BiLSTM performs best in classification and complexity (95.7% 70–30% split; 90.3% LOSO). RCNN performed similarly (95.9%; 89.2%) with a positive trend with subject numerosity. DeepConvLSTM achieves similar classification performance (95.2%; 90.3%) but requires ×57.1 and ×31.3 more Multiply and ACcumulate (MAC) and ×100.8 and ×28.3 more Multiplication and Addition (MA) operations, which measure the complexity of the network’s inference process, than BiLSTM and RCNN, respectively. The BILSTM and RCNN perform close to DeepConvLSTM while being computationally lighter, fostering their use in embedded systems. Such lighter algorithms can be readily used in the automatic ergonomic and biomechanical risk assessment systems, enabling personalization of risk assessment and easing the adoption of safety measures in industrial practices involving MMH.

## 1. Introduction

Human Activity Recognition (HAR) is the task of classifying agents’ actions based on sensor data. The knowledge of human behaviors has potential applications in many fields, such as ambient-assisted living [1], rehabilitation [2], industrial tasks safety [3], and Human–Robot Interaction (HRI) [4]. In the view of Industry 4.0, smart automation is permeating traditional industrial practices, aiming at increasing productivity while improving operators’ working conditions. In this context, humans and robots collaborate on the same tasks in a shared workplace [5], and Manual Material Handling (MMH) activities are expected to remain dominant in many industrial fields. These occupations expose workers to high biomechanical risks that frequently induce Work-related Musculoskeletal Disorders (WMSDs) [6]. Therefore, the automation of the WMSD risk assessment is the key to guaranteeing workers’ health.

To this end, HAR algorithms, determining which action led to specific measures in the sensor data streams, allow for automatizing the workplace ergonomics and biomechanical evaluation processes [7]. Sensor data classified by HAR algorithms can come from external devices, such as cameras [8], or wearable sensors. The latter are generally preferred because of their flexibility, portability, and unobtrusiveness [9,10]. In particular, Inertial Measurement Units (IMUs) are commonly used since they are small, affordable, and available in many commercial goods. Superficial Electromyography (sEMG) signals are valuable complements of IMUs, as they provide many parameters regarding muscle contractions and contain information on the movement intentions of the subject wearing the device [11]. Moreover, sEMG signals allow for recognizing movements [12], evaluating muscle forces, and predicting handled loads [13], desirable features for both ergonomic assessment [14] and robot control in human–robot shared tasks [15,16].

Many Machine Learning (ML) techniques, such as Support Vector Machine, K-Nearest Neighbor, Hidden Markov Models, Gaussian Mixture Model, Decision Tree, and Bayes Network, have been employed in HAR [17]. One of the main challenges that ML techniques need to face is the complexity of the extraction of the more discriminative features, especially because various actions can be very similar to each other (inter-activity similarity), the same action can be performed differently from person to person (intra-activity variability), the sensor types and their placements highly influence the data streams obtained (sensor variability), and often the activities can be considered as composite or concurrent. Despite the notable evolution of ML methods, there is no universal approach for selecting the best feature set representing human activities, and this process is highly demanding and time-consuming. To solve this issue, Deep Learning (DL) methods have the potential to model high-level abstraction from complex data, reducing the workload of feature selection [10]. In HAR applications, DL proved its ability to solve the problems of intra-activity variability and inter-activity similarity [18]. Various deep neural network structures have been used to highlight different characteristics of the input sequences [19,20]. The most common architectures employed with wearable sensor inputs include Convolutional Neural Networks (CNNs) [21]; Recurrent Neural Networks (RNNs) [22]; Feedforward Neural Networks (FNNs), such as autoencoders [23]; and their combinations [24]. In recent years, some researchers investigated some more recent models, such as Transformer [25], Temporal Convolutional Network (TCN) [26], Inception modules [27], and attention mechanism [28]. However, these latter solutions do not significantly improve classification performance, even when they are fused together. We believe that the increased complexity is not worth the performance improvement, especially considering high-impact applications in which HAR should be performed online on embedded systems.

Nowadays, most ML and DL research applied to HAR regards locomotion or Activities of Daily Living (ADLs) [29]. State-of-the-Art (SoA) HAR studies in industrial applications mainly focus on assembling and packaging activities [30], whereas only a few works regard MMH activities. In addition, most of these works adopt ML methods [31,32] instead of DL. This may be due to the scarcity of available datasets regarding MMH activities [33], which can provide a significant amount of data to let DL methods avoid overfitting. Therefore, we propose to explore DL algorithms’ potential in achieving a highly accurate HAR in MMH to make it reliably usable both for biomechanical overload risk assessment and human–robot shared tasks. The main contributions are hence a comparison of DL algorithms on an MMH dataset that is suitable for both goals and the selection of the best algorithm in terms of trade-off between performance and complexity.

In this work, we used the fully labeled dataset of wearable sensor data (kinematic and sEMG data), acquired during MMH activities [34], to compare DL frameworks in the ability to recognize MMH activities. In particular, we considered four network models:

1. BiLSTM;
2. Sparse Denoising Autoencoder (Sp-DAE);
3. Recurrent Sp-DAE;
4. Recurrent CNN (RCNN).

For each model, we first evaluate the effects of different hyperparameters on the network’s classification performance in addressing the HAR of MMH activities problem and select their best combination. Then, we compare the classification ability of the different network frameworks and evaluate the performance of the best network architectures adopting the Leave-One-Subject-Out (LOSO) validation technique. Finally, we compare the selected neural networks with the DeepConvLSTM [35], a recurrent network already used as a benchmark [36], in terms of recognition ability and computational complexity.

The paper is organized as follows: Section 2 presents the background, including the descriptions of the DL approaches most used in HAR; Section 3 presents the input data along with their processing steps and the proposed network architectures; Section 4 presents the results obtained; Section 5 discusses the results; and finally, Section 6 closes the paper.

## 2. Background

### 2.1. Recurrent Neural Network

RNNs are designed to model time series data. They are composed of hidden units (HUs) connected with a feedback loop that passes the previous hidden state information to itself with a certain delay and weight, providing a memory to the network. In this way, the network’s output depends on the current and the previous inputs. Traditional RNN structures are not good at learning temporal relations on long time scales because of the vanishing gradient problem [37]. This can be solved by the Gated Recurrent Unit (GRU) [38] or LSTM unit [39]. They implement gating mechanisms, creating paths through time where the gradient can flow, and consequently, longer sequential time series can be modeled. These RNNs compute the current output considering only the previous information. However, continuous human movements can be better predicted if successive states are considered. Therefore, BiLSTMs [40], considering the input both in forward and backward directions, provide more information that can improve the network performance in classifying time sequences. These characteristics make RNN architectures particularly suitable for HAR applications. Murad and Pyun [41] proposed one of the first uses of LSTM networks for HAR. They tested unidirectional, bidirectional, and cascade architectures on some benchmark datasets, one regarding assembling activities, and they found that LSTM models outperformed both the ML approach and CNN architectures. Porta et al. [42] employed a BiLSTM network to classify MMH activities, comparing the classification accuracy using a single IMU and the Full-Body (FB) configuration. Arab et al. [43] compared four deep neural networks to classify 10 logistic-oriented activities using three IMUs placed on different body positions and found that BiLSTM network structures reach higher performances than the Feedforward Neural Network (FFNN) and CNN.

### 2.2. Autoencoder

Autoencoders are a fascinating architecture because of their unsupervised characteristic. They are commonly used as a pre-training method for feature extraction and dimensionality reduction in DL networks. They are FFNNs composed of an encoder and a decoder module. The first encodes the inputs in a latent space, and the latter decodes the hidden feature representation back to the original signals. The input and output layers have the same number of nodes; instead, the hidden layer, having fewer neurons, allows for obtaining a low-dimensional feature set that can be used as input of a classification layer. Autoencoders are used individually [44] or in Stacked Autoencoder (SAE) architectures in which autoencoders are the building blocks. Almaslukh et al. [45] applied an SAE to publicly available datasets to enhance recognition accuracy. They stacked two autoencoders and a softmax layer to classify data generated by the sensors embedded in the smartphone. Vincent et al. [46] tried out a modified version of an SAE, the Stacked Denoising Autoencoder (SDAE), to extract more robust features from corrupted data. Gu et al. [47] used the SDAE stacked with a softmax layer to classify smartphone sensors’ data for Locomotion Activity Recognition. The SDAE has been used as a feature extraction method in HAR for ADLs, but its ability to recognize MMH activities has not been investigated yet.

### 2.3. Convolutional Neural Networks

Deep CNNs have been applied to the recognition of ADLs with good performances [48], and, in recent years, some researchers have applied them to MMH activities. CNNs include a variable number of stacked hidden layers that can hierarchically extract characteristic features representing temporal and spatial dependencies. The first layers detect local connected features (*feature maps*) performing convolution operations on the raw sensor data through filters with shared weights; then pooling layers (often *max-pooling*) fuse similar features reducing the number of the parameters and introducing scale invariance; fully connected layers build stronger features; and finally an inference layer, composed of a *softmax* function and a cross-entropy loss function, classify the input. Since time series have one dimension, some researchers used 1D-CNNs to capture local dependencies along the temporal dimension. Yoshimura et al. [36] used the acceleration data of a sensor placed on the worker’s wrist and compared six DL methods to classify picking and packaging activities. Arab et al. [43] compared the single-sensor modality with two different sensor fusion techniques and found that 1D-CNN underperforms compared to BiLSTM networks with multisensory input. Indeed, since multiple sensor signals are generally available, extracting spatial correlation among the different sensor data, besides the temporal dependencies, is advisable. Niemann et al. [49] vertically stacked motion data and used a temporal 2D-CNN to classify seven handling activities, and Syed et al. [50] compared four CNNs on IMU data to recognize warehouse activities. However, CNNs underperform at extracting long-term dependencies compared to RNNs. Thus, they are frequently combined with other architectures with complementary modeling functionalities.

### 2.4. Hybrid Networks

Deep hybrid networks attract the attention of many researchers aiming at exploiting the different abilities of the various network architecture models.

#### 2.4.1. Recurrent Convolutional Neural Networks

One of the most interesting combinations is a CNN plus RNN to extract local and long-term dependencies in the time series data. Different research groups combined a CNN with LSTM in various ways and obtained good performances on publicly available ADL datasets [51,52]. Ordóñez and Roggen [35] were the first researchers to propose a CNN-LSTM architecture (DeepConvLSTM) for HAR of assembling activities, proving their higher performances compared to both shallow and deep CNNs. More recently, He et al. [52] developed a model that integrates a temporal CNN, a bidirectional GRU, and an attention mechanism to classify ADLs. Yoshimura et al. [36] used a DeepConvLSTM network as a benchmark to compare their architecture’s performance to classify MMH activities in a logistics environment. Thus, further investigation into the ability of RCNNs to classify MMH activities is needed.

#### 2.4.2. Recurrent Autoencoder Networks

Even if autoencoders showed a high aptitude to extract low-dimensional features and denoise raw sensor data, few studies combine them with other architectures in wearable sensors-based HAR. Gao et al. [53] proposed an HAR algorithm based on inertial data of smartphones composed of a Stacking Denoising Autoencoder (SDAE) and a LightGBM (LGB) classifier. They compared its performance with those of a single SDAE, XGBoost, and CNN concluding that the SDAE-LGB combination reaches higher accuracy and is more generic and robust than the other algorithms. Li et al. [54] compared three different unsupervised learning techniques, the Sparse Autoencoder (SpAE), the DAE, and PCA, for HAR based on accelerometer and gyroscope sensor data. They found that the SpAE performed better than the others. However, as for autoencoders, to the best of our knowledge, no researchers have investigated the ability of recurrent autoencoder networks to recognize MMH activities.

### 2.5. Selected Architectures

Among the aforementioned network architectures, some are more suitable for HAR, giving more chances to obtain higher recognition accuracies on MMH activities. The BiLSTM outperforms other RNNs when dealing with time sequences, and DAE is generally able to extract more robust features in an unsupervised manner. Thus, it is interesting to explore both their single implementations and their combination. In addition, since CNNs proved their ability in feature extraction for classification tasks, but lack in detecting long-term dependencies in time series data, the CNN-RNN combination is a promising option for HAR. Therefore, the network architectures to classify MMH activities proposed in this study are the BiLSTM, a Sp-DAE, a Recurrent Sp-DAE, and an RCNN.

## 3. Dataset and Methodology

### 3.1. Dataset and Networks Input

The public MMH dataset used in this work has been presented in our previous paper [34]. It has been collected on 14 subjects in a laboratory environment and comprises 2 activity sets: an MMH activity set and an isokinetic activity set. The former contains lifting and carrying activities of different loads placed at various heights and performed in bimanual and one-handed modes. The latter includes one-handed load lift actions of different weights and at different velocities. In particular, the seven activities included in the dataset are as follows: N-pose (N), Lifting from the Floor (LF), Keeping lifted (K), Placing on the Table (PT), Lifting from the Table (LT), carrying (W), and Placing on the Floor (PF). This dataset includes labeled FB motion data measured by the commercial device Xsens MVN suit (Xsens Technologies B.V., Enschede, Netherlands) and sEMG signals of arm and forearm muscles measured with a device previously developed by our team [55]. The wearable inertial system is composed of 17 motion trackers attached with elastic straps to the human body segments. Each tracker contains a 9-axis IMU to acquire kinematic data, which are processed by the proprietary software fusion engine that guarantees high robustness against magnetic disturbances and performs the data processing to provide 3D positions, orientations, and angular velocities of all the 23 segments and the 3D angles of the 22 joints. The sEMG data are filtered both on the hardware implemented on the device we developed and with software filters, a Notch filter at 50 Hz, a Butterworth bandpass filter between 20 and 500, and then the sEMG signals are rectified and normalized relative to the subject-specific Maximum Voluntary Contraction (MVC). The RMS is calculated with the envelope over a 250 ms window. As presented in our previous work [34], inertial and sEMG data are both provided with a timestamp which allows for their synchronization.

In this work, we adopted an input composed of 10 signals: eight motions and two sEMG signals. Selected motion data can be gathered with other sensing technologies to enhance the generalizability of the proposed approach. These include the flexion of the chest, shoulder, elbow, and knee; the hand position and acceleration in the front direction relative to the pelvis; the velocity on the transversal plane; and the position on the global vertical direction of the pelvis. The Root Mean Square (RMS) sEMG of the biceps brachii and the brachioradialis muscles complete the input. The sEMG signals were included as they can provide insights on held loads and muscular effort that are useful for both biomechanical overload estimation and robot control in human–robot shared tasks [56]. Moreover, multiple sensing modalities can provide higher performances in HAR, since they supply complementary information to recognize actions [19]. When using multimodal data, a challenging task is the choice of the sensing modality fusion technique. Multisensory fusion is usually classified into three categories: Early-Fusion (EF), Mid-Fusion (MF), and Late-Fusion (LF) [57]. EF, also called data fusion, combines the inputs collected by multiple sensors at the beginning of the training phase, allowing for the enhancement of the classification system’s generalization and reliability. This approach gives the best results when the sensors involved measure the same physical phenomenon. However, when the input data are acquired with heterogeneous devices, MF and LF should be preferred. MF makes a new feature vector input for the classification step, combining the features extracted from the different sensors, and it is commonly used to quantify the distinction between measured data signals. LF employs classifier ensembles combining the classifiers’ outputs. In this study, we adopted the EF technique, vertically stacking the data into a 2D matrix because the data are mostly homogeneous, since only two out of ten inputs are not motion data.

After input data preprocessing, the time series are segmented by a sliding window of a fixed length to obtain data segments as inputs for the HAR algorithm. Ideally, each segment should contain one activity; therefore, the segment length is key to optimizing the recognition accuracy. Wider windows contain more information about the activity but increase the chance of having an activity transition inside the windows. On the contrary, narrower windows increase the risk of not having enough information about the activity. Various research groups face this issue. Most of them attempt fixed window sizes [47], while others use random length and position frames [58]. In this study, we found a good compromise using short intervals of 1 s length with 1 s stride and no overlap. This also allows for the implementation of light networks, thus limiting the computational cost and making the network portable.

The class imbalance issue must be tackled [59]. The network tends to better classify classes with many training samples and ignore those with fewer examples. This is a common issue when dealing with short activities such as falls. To face this problem, we down-sampled the largest class (N-pose) and augmented the classes with fewer examples. In particular, we replicate the class segments, adding white Gaussian noise to prevent overfitting and enhancing the models’ robustness. This data augmentation technique proved its effectiveness in many applications, ranging from image classification [60] to HAR in manufacturing [61].

### 3.2. Network Architectures

#### 3.2.1. BiLSTM

**Figure 1:**
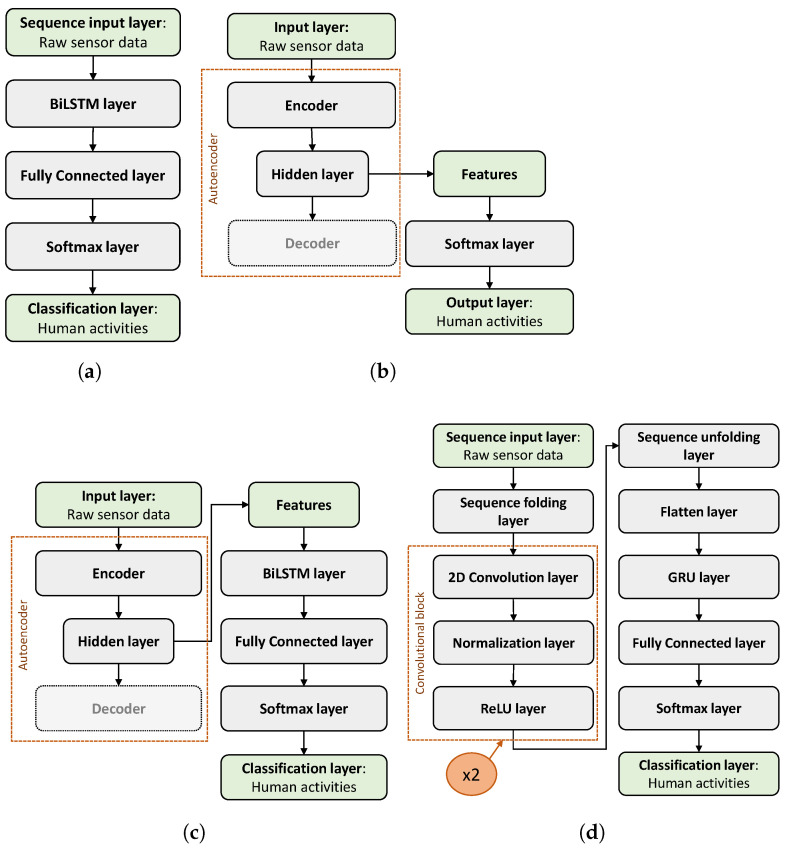
The graphical representation of the network architectures: (**a**) BiLSTM; (**b**) Sp-DAE; (**c**) Recurrent Sp-DAE; and (**d**) RCNN.

The BiLSTM is the simplest architecture proposed. As shown in Figure 1a, it consists of 5 layers. First, the sequence input layer has a size equal to the number of inputs (10), and then the input is fed into the BiLSTM layer with a variable number of HUs and maximum number of epochs (EpS). The output feature set produced by the BiLSTM layer is then provided to the fully connected layer that learns non-linear combinations of these features. Then, the softmax layer computes the probability distribution over the predicted output classes, and the classification layer computes the cross-entropy loss for the classification with mutually exclusive classes. We performed 50 trainings to find the best three Eps-HUs number pairs. The values of the hyperparameters used in these trainings are summarized in Table 1.

#### 3.2.2. Autoencoder

The autoencoder implemented is a Sp-DAE (Figure 1b). It consists of three layers and is trained to reconstruct the original raw sensors’ data, with a variable number of HUs and Eps. After the first unsupervised training, the decoder is discarded and the learned features are fed into a softmax classifier that computes the cross-entropy loss in a supervised fashion. To select the best three Eps-HUs number pairs of the Sp-DAE, we performed 128 trainings. The values of the hyperparameters used in these trainings are summarized in Table 2.

#### 3.2.3. Recurrent Sp-DAE

The Recurrent Sp-DAE (Figure 1c) is implemented by stacking the BiLSTM architecture (Section 3.2.1) on the encoder (Section 3.2.2). In this way, the BiLSTM, learning the temporal dependencies, should provide better recognition performances than the single Sp-DAE. We performed 48 trainings in two steps to select the optimal Sp-DAE and BiLSTM architecture combinations. First, we used the best three Eps-HUs pairs selected for the BiLSTM and the Sp-DAE. Then, we added five more BiLSTM hyperparameter combinations: 300 HUs and from 3000 to 5000 Eps with 500 increments.

#### 3.2.4. RCNN

Lastly, the proposed RCNN (Figure 1d) consists of 14 layers: the sequence input layer; the sequence folding layer; two convolution blocks that in turn are composed of three layers: the 2D convolution layers, the normalization layer, and the ReLU layer; the sequence unfolding layer; the flatten layer; the GRU layer; the fully connected layer; the softmax layer; and the classification layer. The sequence input layer, different from the BiLSTM network (Section 3.2.1), has a 2D input size equal to the number of inputs per sample number of the windows (10 × 240). Thus, the segmented time series inputs are considered as images. Then, the sequence folding layer, converting the sequences of images to an array of images, allows for applying convolutional operations independently to each time step. As a result, after the convolution block operations, the sequences are restored by the sequence unfolding layer. The 2D convolution layer applies sliding local receptive fields (*filters*) to the 2D input and thus learns the local features on the regions covered, reducing the model size compared to the 1D filters. In this study, we used filters with the most popular size choice of 3 × 3 for both 2D convolutional layers. Aiming at selecting the best-performing RCNN structure and hyperparameters, we performed various trainings to evaluate the classification performances obtained with different numbers of convolution blocks and hyperparameter combinations. In particular, we compared the performances obtained with two and three convolution blocks and we explored different number of filters: 2, 4, 8, 16, 32, 64; dilation factors: 1, 2, 4; stride: 1, 2, 3, 4; and padding: 0, 1. Thus, we selected the RCNN structure with two convolution blocks, we set the filter number to 32 and the padding to 0 for both the 2D convolutional layers, and we left the default values both for the stride (1 × 1) and the dilation factor (1 × 1) for the first convolutional layer, but we set the stride to 1 × 4 the dilation factor to 2 × 2 for the second convolutional layer.

The use of a dilation factor allows for the exponential expansion of the receptive field without changing the size of the field map and without the need for pooling layers that cause information losses in the feature representation, since they down-sample the feature maps. After the convolutional layer, we used an instance normalization layer that normalizes the data for each observation independently and, compared to the batch normalization layer that normalizes each channel independently, improves the training convergence, besides reducing the sensitivity to network hyperparameters. Then, the ReLU layer speeds up learning compared to sigmoid or tanh non-linear functions. After the two 2D convolutional blocks and after the sequences are restored by the sequence unfolding layer, the flatten layer converts the images to feature vectors that are fed as input in the recurrent layer. In this architecture, after having compared the performances obtained with the GRU and BiLSTM layers, we found out that they have comparable recognition performances. Thus, we chose to use the GRU layer because it limits the network complexity. Finally, the fully connected layer, the softmax layer, and the classification layer, operating on the extracted features, determine the human activity classes. We performed 25 trainings to select the best three Eps-HUs pairs. The hyperparameters used in the implementation of the RCNN are summarized in Table 3.

### 3.3. Networks Training and Testing

The training and testing of the networks followed three steps. The first aimed at selecting two architectures out of four with the best classification performance. In the second step, we analyzed the performance trend of the best two networks with an increasing number of subjects (1 to 14) to evaluate whether classification performance stabilizes or it might increase with a larger dataset. Here, we also selected the best one out of three parameter combinations for the two architectures previously selected. Then, we adopted the Leave-One-Subject-Out (LOSO) technique to assess the classification performances obtained training the two networks with 13 different datasets with increasing subject numerosity (1 to 13) and testing on the fourteenth subject data not considered during training. The latter two steps were applied to the DeepConvLSTM network (Section 2.4.1), used as a benchmark in terms of both classification performance and computational complexity.

More in detail, in the first step, for each network model previously depicted (Section 2), we ran a large amount of trainings to select the best hyperparameters for each structure. In particular, we investigated the best number of Eps and HUs that trigger higher performances. The best three Eps-HUs pairs were estimated with the grid search approach, optimizing the classification performance. For every test, we considered the dataset composed of all 14 subjects, and we took 70% of the dataset for training and the rest 30% for testing. For the second step, we organized the data in 14 different datasets composed of an increasing number of subjects, and also in this case, for each dataset, we considered the 70% for training and 30% for testing. In addition, for each of the three hyperparameter pairs selected, we performed three different trainings for each subject numerosity, for a total of 126 trainings, to evaluate the mean and standard deviation of the F1-scores obtained.

The training and testing are performed in Matlab, using the Deep Learning Toolbox^TM^ (R2021b, MathWorks, Natick, MA, USA) that provides a framework to design and implement deep neural networks, on a machine with an Intel i9-12900K CPU @ 3.20 GHz, 64 GB RAM, and an NVIDIA GeForce RTX 4090.

#### Metrics

Many performance assessment metrics to compare the aforementioned architectures are available: the accuracy, the precision, the recall, the F1-score, the confusion matrix, and the Area under the ROC curve [10]. Among them, the most used are the accuracy and the F1-score. In this study, we used the F1-score because it is generally more trustworthy than the accuracy when having a class imbalance problem. Thus, it helped us to countercheck that this issue had been solved. In particular, among the three different F1-score types, macro, micro, and weighted, we used the macro F1-score because it considers all the classes equally, regardless of how many samples each has. Thus, if there are still classes with less samples, this index decrease significantly and a misclassification problem arise. Also, a weighted F1-score accounts for class imbalance, but giving more weight to larger classes does not have the same power as a macro F1-score to check if class imbalance has been solved. Even worse is the micro F1-score that is computed across all classes and can be used only when classes are definitely balanced.

The computational complexity can be assessed with different metrics, but no “universal” measure exists [62]. In this study, we used the number of Multiply and ACcumulate (MAC) and the total number of Multiplication and Addition (MA) operations because they allow us to evaluate the memory usage and the computational cost of the network for a large variety of computing architectures. MAC and MA are computed over the complete inference process of one input, from when it is provided to the network up to the classification output. In addition, we also reported the number of Learnable Parameters (LPs) (weight and biases), the memory (MB) required to store all the LPs and the estimated inference latency considering an $UP^{2}$ board (AAEON, New Taipei City, Taiwan R.O.C) with a CPU Intel Atom^TM^ X5-E3940, and 4 GB LPDDR4 RAM (1066 MHz; 16 bit bus width) as the embedded platform. The inference latency is computed as the ratio between the total operations needed for the network and the attainable operations reduced by an empirical efficiency factor (0.7) to account for kernel inefficiency.

## 4. Results

### 4.1. Best Parameters Selection

**Figure 2:**
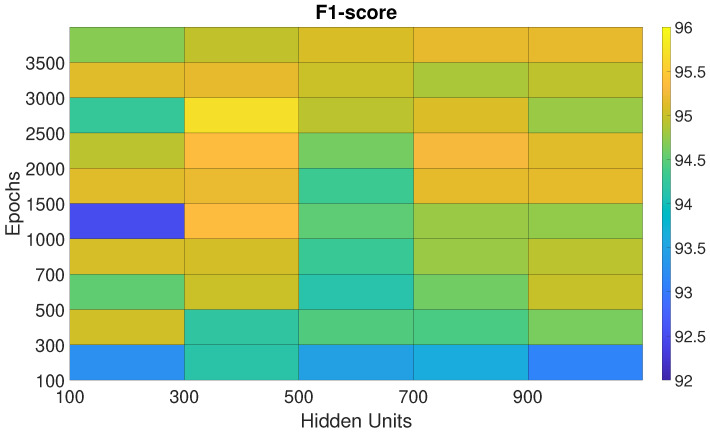
F1-score of the BiLSTM networks trained with different epoch–hidden unit pairs.

**Figure 3:**
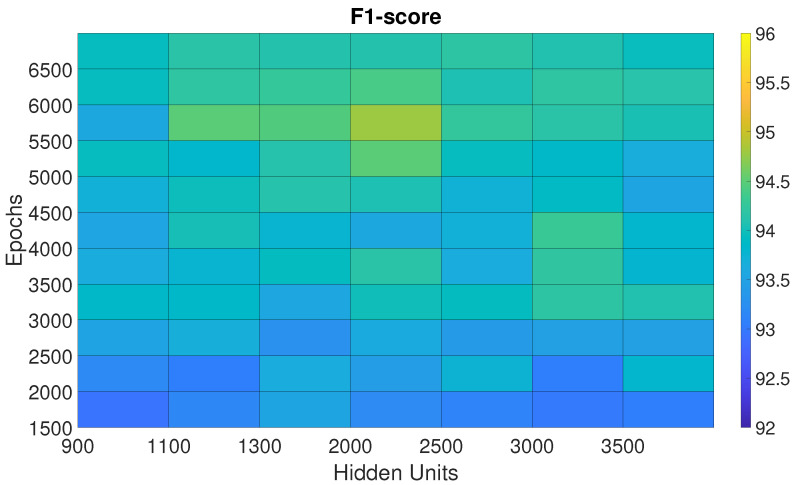
F1-score of the Sp-DAE trained with different epoch–hidden unit pairs.

**Figure 4:**
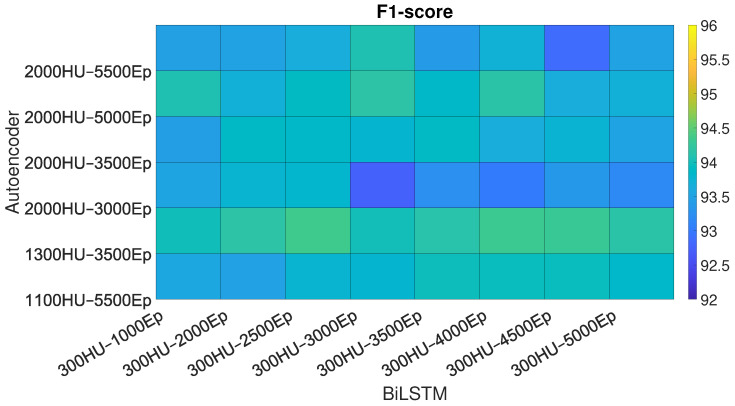
F1-score of the Recurrent Sp-DAE trained with different Sp-DAE-BiLSTM combinations. For each network, we reported the Hidden Unit (HU)–Epoch (Ep) pair on the axis.

**Figure 5:**
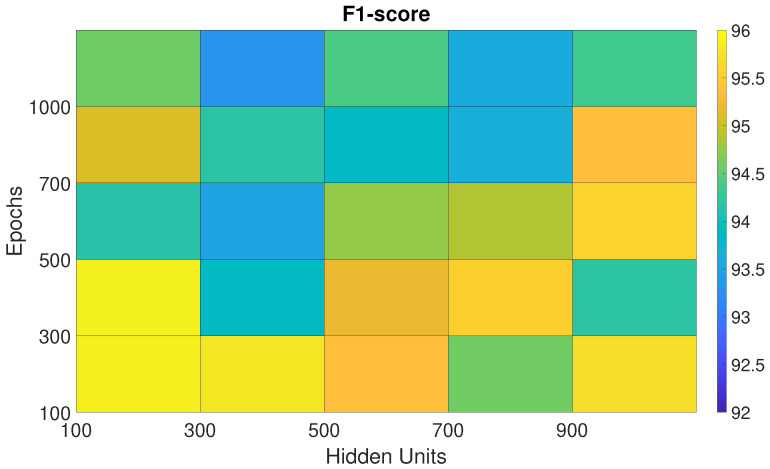
F1-score of the RCNN trained with different epoch–hidden unit pairs.

The performances of the BiLSTM, Sp-DAE, and RCNN architectures, obtained with the different Eps-HUs pairs, are reported as checkerboard plots in Figure 2, Figure 3, and Figure 5, respectively. Each cell corresponds to the F1-score obtained by a network with a specific Eps–HUs combination. Indeed, we reported Eps and HUs on the vertical and horizontal axes, respectively. Figure 4 shows the F1-scores obtained by the Recurrent Sp-DAE with different BiLSTMs-Autoencoders combinations, which are reported on the horizontal and vertical axes, respectively. Figure 2, Figure 3, Figure 4 and Figure 5 have a color scale of the F1-score on the right side, for which the limits (from 92% to 96%) are consistent across them. This visualization eases the observation of the best-performing Eps-HUs pairs or BiLSTMs-Autoencoders combinations in terms of the F1-score and how moving from these values deteriorates the network scores.

#### 4.1.1. BiLSTM

The BiLSTM network classification performance converges to the maximum with 300 HUs and different values of Eps (Figure 2). The best Eps-HUs pair is 300 HUs–2500 Eps with 95.7%, followed by 300 HUs–2000 Eps and 300 HUs–1000 Eps with 95.3%.

#### 4.1.2. Sp-DAE

In Section 3.2.2, we introduced a large number of combinations of Eps-HUs pairs for the Sp-DAE. For the sake of clarity, we report only the subset of parameters that performed better, excluding combinations that provided limited F1-scores (Figure 3).

The Sp-DAE shows a stronger convergence in the middle of the checkerboard plot area. The best Eps-HUs pair is 2000 HUs–5500 Eps with 94.7%, followed by 1100 HUs–5500 Eps and 2000 HUs–5000 Eps with 94.5%. Although the hyperparameters of Sp-DAE are strongly higher than BiLSTM, the loss of one percentage point of the F1-score compared to the BiLSTM suggests that the Recurrent Sp-DAE could improve the classification performance.

#### 4.1.3. Recurrent Sp-DAE

As reported in Section 3.2.3, we trained 48 combinations of Sp-DAE and BiLSTM. Figure 4 shows the checkerboard plot of the F1-scores obtained, and the Sp-DAE with 1300 HUs and 3500 Eps showed to be the best in extracting input features for BiLSTM. The best BiLSTM has 300 HUs and 2500 Eps followed by 4000 and 4500 Eps. All these Recurrent Sp-DAEs have F1-scores close to 94.3%. Different from SP-DAE, where the softmax layer better recognizes the different activities with features extracted by encoders with higher dimensions, in the Recurrent Sp-DAE, the features extracted by an encoder with smaller Eps and HUs perform better.

#### 4.1.4. RCNN

Figure 5 shows the F1-scores of the RCNN trained with the proposed Eps–HUs pairs. Different from the BiLSTM and the Sp-DAE networks, the RCNN exhibits higher classification performances with lower HUs and Eps, keeping the F1-scores at the same level of the BiLSTM network. The best Eps–HUs pair is 100 HUs–300 Eps with 95.9%, followed by 100 HUs–100 Eps with 95.8% and 300 HUs–100 Eps with 95.7%.

### 4.2. Network Architecture Comparison

Training outcomes of the four different neural networks show that the Sp-DAE underperforms compared to the other two network architectures, regardless of the BiLSTM layers. The Sp-DAEs are indeed stuck at an F1-score of more than one percentage less than the other architectures. Therefore, BiLSTM (95.7%) and RCNN (95.9%) are preferred to recognize MMH activities exploiting wearable sensor data and are further analyzed.

**Figure 6:**
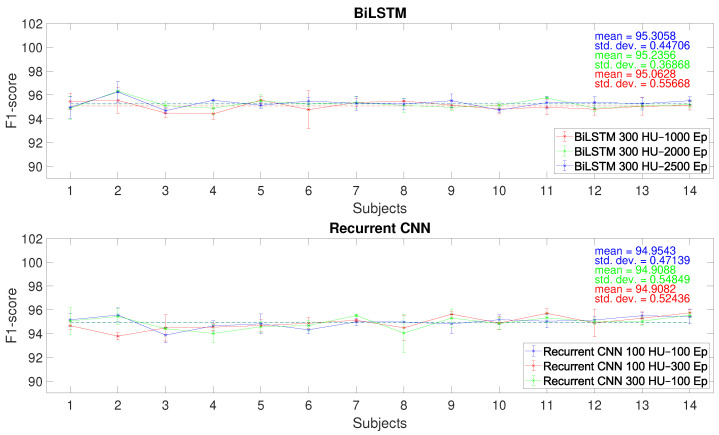
F1-score for each subject numerosity of the different architectures with the best Hidden Unit (HU)–Epoch (Ep) pairs.

Figure 6 shows the F1-scores of these networks with the best three Eps-HUs pairs while varying the number of subjects in the training dataset. Dotted lines represent the overall average of the F1-score. Regarding the BiLSTM, all three hyperparameter pairs show great recognition performances, and, since the network complexity does not change, we decided to use the 300 HUs–2500 Eps pair for the LOSO comparison (Section 4.3) since it has a higher F1-score when trained with 14 subjects. In addition, the trend of the average F1-score shows a convergence to 95% with increasing subject numerosity and does not drop under the 95% threshold from the 11th subject. The RCNNs show almost the same overall means (94.9%). Thus, we used the 100 HUs–300 Eps pair for the LOSO comparison (Section 4.3), since it has a lower complexity and higher F1-score when trained with 14 subjects. In this case, the average F1-score shows a slightly increasing trend with increasing subject numbers.

### 4.3. Performance of the Selected Networks with LOSO Validation

**Figure 7:**
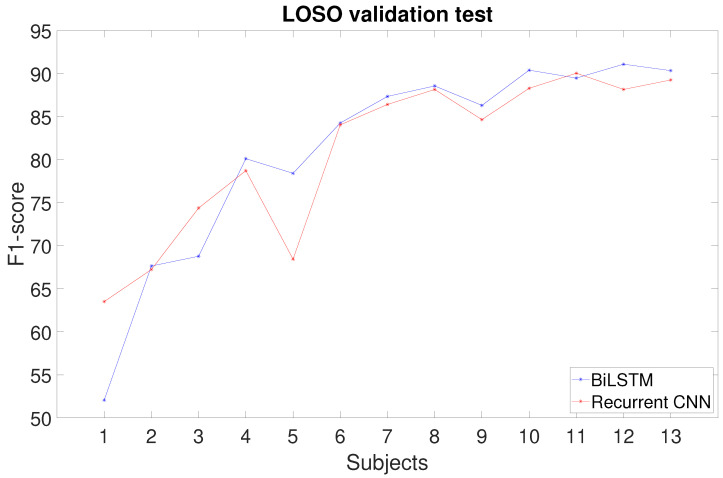
F1-score for each subject numerosity with the LOSO test of the (**a**) BiLSTM with 300 hidden units and 2500 epochs and (**b**) RCNN with 100 hidden units and 300 epochs.

In the LOSO validation, we considered the BiLSTM with 300 HUs–2500 Eps and the RCNN with 100 HUs–300 Eps trained with subject numerosity ranging from 1 to 13. Figure 7 reports the F1-scores obtained for the two network architectures. Both show a convergent trend, but the maximum value, reached with 13 subjects, is 90.3% for the BiLSTM, while the RCNN stopped one percentage point below (89.2%).

**Figure 8:**
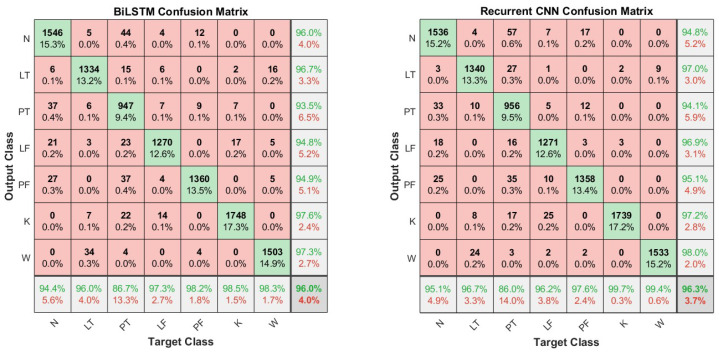
Confusion matrices of the test data for the BiLSTM and RCNN network architectures trained with the 70–30 split mode.

The F1-scores for each activity class obtained in the LOSO validation, considering 13 subjects in the training dataset, are reported (Table 4). The score is higher than or close to 90% for each class, except for the PT actions. This is confirmed by the results presented in the confusion matrices in Figure 8, where the PT actions are sometimes misclassified as N-pose action.

### 4.4. Comparison of the Selected Networks with SoA

Table 5 reports the F1-score, accuracy, precision, MAC and MA operations, number of the LP, memory (MB) required to store all the LP, and inference latency of the BiLSTM with 300 HUs–2500 Eps, RCNN with 100 HUs–300 Eps, and the DeepConvLSTM with 128 HUs as reported by Ordóñez and Roggen [35] and 300 Eps as per our RCNN. The network classification performances are reported for the 70–30% split of the complete dataset and for the LOSO validation.

## 5. Discussion

The results presented in Section 4 show that the BiLSTM and RCNN perform better than networks based on autoencoders, though being simpler in terms of parameters and epochs needed to achieve peak performance. Sp-DAE, even if stacked with the BiLSTM architecture, reported the worst classification performances and achieved higher scores only by increasing the HU number up to 2000. Surprisingly, adding the BiLSTM deteriorated the algorithm performance compared to softmax, suggesting that the extracted features canceled the potential benefits of time recursion.

In the 70-30 split training, the BiLSTM and RCNN performed similarly, but the RCNN showed an increasing trend of the F1-score as the number of subjects in the dataset increased, suggesting that it could result in higher classification performances with more subjects. However, the addition of convolutional layers notably increases the network complexity compared to the BiLSTM. In the LOSO analysis, the BiLSTM achieved a better F1-score, though being simpler than the RCNN. Therefore, for this dataset, the BiLSTM proved to be the best choice both in terms of classification performance and network complexity. However, the RCNN, having an F1-score close to BiLSTM’s, and showing a slightly increasing trend of the F1-score with the subject number, could potentially perform better than the BiLSTM with a higher number of subjects. In addition, the possible improvement might justify the bigger complexity of the network and the consequent computational burden, but this trend should be confirmed and evaluated with a larger dataset.

The LOSO validation confirmed that both the BiLSTM and RCNN have the potential to achieve high F1-scores when data comes from a new subject, increasing the reasons to further investigate in this direction. This result is consistent with a similar BiLSTM architecture that has been implemented in [42] to classify MMH tasks. The authors obtained comparable F1-scores (91%) to ours but at the cost of using the FB configuration, composed of the three-axis accelerations and angular velocities of all the 17 IMUs of the Xsens suit, resulting in 102 network inputs. This makes their network more complex and the method less flexible and generalizable since the users are forced to wear an FB suit equipped with IMUs to have suitable motion data. Conversely, the proposed BiLSTM network is much lighter, as the input is composed of only 10 variables. Moreover, using joint angles, velocity, and acceleration data opens the way to test and enhance the network with motion data gathered with other technologies, such as RGB camera-based motion capture. This is a crucial point for HAR because the purchase and use of an FB suit can be unaffordable in MMH activities. Indeed, nowadays, many researchers exploit signals acquired with IMUs embedded in smartphones or even smartwatches for HAR of ADLs [63] and even MMH activities [64] but still with low classification performances.

To the best of our knowledge, the proposed RCNN has never been used for HAR, as the existing RCNN is much more complex. DeepConvLSTM [35] has four convolutional layers instead of our two layers and uses two LSTM layers instead of one GRU. The comparison with our proposed networks showed that DeepConvLSTM has similar (even lower) recognition performance despite its consistently higher computational cost. This may be interpreted with an unnecessary complexity of this network in relation to the classification task. This is confirmed by the application of this network in other datasets [36,64,65]. Considering the supposed target applications, the classification performance achieved on most classes allows for using the proposed network. The most critical class was PT (placing the object on the table), which is mainly confused with the N-pose static position, maybe due to its little variation in joint flexions, especially when handling light loads (Figure 8). In the evaluation of the biomechanical overload due to MMH, which causes WMSDs, newly developed methods rely more and more on quantitative data instead of visual inspection of videos [14,66]. In our previous work [14], we proposed a system for a quantitative evaluation of the ergonomic risk indices in a challenging outdoor scenario, and we demonstrated the consistency and time effectiveness of our system compared to the traditional evaluation method. However, the segmentation of the recorded time series is manually operated by a rater and is highly time consuming. Thus, both the proposed HAR networks, enabling the automatic segmentation, drastically reduce the time that the rater needs for manual segmentation. Nevertheless, if higher recognition performances are desired, the raters can review the network output and correct it, providing valuable new data to train the network and increase its classification ability. The lightness of the selected network, especially the BiLSTM, which has an incredibly low latency (76 μs), makes this activity segmentation easily applicable to real-time applications when shared human–robot tasks are concerned. Indeed, HAR proved to be a fundamental component in human–robot collaboration already in Industry 4.0 [67], especially to plan the behaviour of robots [68], and it will feature more in Industry 5.0, which foresees a seamless collaboration of humans and robots in industrial contexts [69].

## 6. Conclusions

This work has been motivated by the scarcity of research on HAR systems for MMH activities, given its potential impact on the automatization of ergonomic risk assessment and human–robot collaboration in shared tasks. In this study, we compared different DL algorithms to classify MMH tasks and proposed the BiLSTM and the RCNN as the best choices. In fact, they achieve a similar (even better) classification performance with regard to the SoA with a significantly lower complexity. BiLSTM is preferable for network complexity, whereas RCNN should be taken into consideration for larger datasets. These networks require a limited set of motion and sEMG data and can be used in ecological settings in both logistics and industry. The abstraction from raw data encourages us to test these networks with motion data gathered with depth/stereo cameras, which would limit the number of wearable sensors and improve the feasibility of their pervasive use in MMH activities performed in spatially limited workplaces. Moreover, we plan to reduce the network input to minimize the sensors that the user needs to wear to obtain segmentation with a comparable F1-score, thus enlarging possible applications of the proposed networks.

## Figures and Tables

**Table 1:** Hyperparameters used in the BiLSTM trainings.

Hyperparameter	Value
Input size	10
Optimizer	Adam
Maximum epochs	100, 300, 500, 700, 1000, 1500, 2000, 2500, 3000, 3500
Hidden units	100, 300, 500, 700, 900
Batch size	128
Initial learning rate	1 × $10^{3}$
Learning rate drop factor	0.1
Learning rate drop period	10
L2 regularization	1 × $10^{-4}$
Loss function	Cross-entropy loss

**Table 2:** Hyperparameters used in the Sp-DAE trainings.

Hyperparameter	Value
Input size	10
Maximum epochs	100, 300, 500, 700, 1000, 1300, 1500, 2000, 2500, 3000, 3500, 4000, 4500, 5000, 5500, 6000, 6500
Hidden units	100, 300, 500, 700, 900, 1100, 1300, 2000, 2500, 3000, 3500
Training algorithm	Conjugate gradient descent
Sparsity regularization	1
Sparsity proportion	0.05
L2 regularization	1 × $10^{-4}$
Transfer function	log-sigmoid
Loss function	Sparse mse

**Table 3:** Values of hyperparameters used in the RCNN trainings.

Hyperparameter	Value
Input size	10 × 240
Filter size	3 × 3
Filter dimension	32
Padding	0
1° CNN layer stride	1 × 1
2° CNN layer stride	1 × 4
1° CNN layer dilation factor	1 × 1
2° CNN layer dilation factor	2 × 2
Maximum epochs	100, 300, 500, 700, 1000
Hidden units	100, 300, 500, 700, 900

**Table 4:** F1-score for each action.

Action	BiLSTM	RCNN
N-pose (N)	89.1%	90.1%
Lifting from the Table (LT)	94.5%	92.4%
Placing on the Table (PT)	84%	85.1%
Lifting from the Floor (LF)	90.3%	88.7%
Placing on the Floor (PF)	88.8%	86.7%
Keeping lifted (K)	92.3%	91.4%
Carrying (W)	93.4%	90.2%

**Table 5:** Comparison among the BiLSTM, RCNN, and DeepConvLSTM. For each network, the F1-scores, accuracies, and precisions obtained with the 70–30 split and LOSO methods; the number of MAC and MA operations; the number of the LP; the memory footprint; and the inference latency are reported.

Parameter	BiLSTM	RCNN	DeepConvLSTM
F1-score 70–30 split	95.7%	95.9%	95.2%
Accuracy 70–30 split	96.0%	96.3%	95.5%
Precision 70–30 split	95.8%	96.1%	95.3 %
F1-score LOSO	90.6%	89.2%	90.3%
Accuracy LOSO	97.2%	96.9%	97.2%
Precision LOSO	91.0%	89.4%	90.3%
MAC	1,492,200	4,212,028	327,027,584
MA	1,532,400	6,972,056	543,762,176
LP	750,607	756,675	15,989,191
Memory (MB)	2.863	2.886	60.994
Latency (ms)	0.076	0.346	7.7

## Data Availability

The data analyzed in the study are openly available in “Manual Material Handling Dataset for Biomechanical and Ergonomics Analysis” at URL https://zenodo.org/records/4633087 (accessed on 24 October 2025).
